# New Publications

**Published:** 1902-09

**Authors:** 


### NEW PUBLICATIONS.

*The Quarterly Bulletin* of the Chicago Veterinary College, published in the interest of the college, has just made its initial bow to the public. It will be continued through the year and sent out among the school's graduates and the profession at large, with a view of promoting the welfare of the college. It will be edited and contributed to by the teaching staff of the college, and give to the profession at large a better knowledge of the work and teaching at that institution. Almost its entire corps of instructors contribute something to the first number. A special announcement of the post-graduate course is added to the first number.

